# Supplementary figures and images for: Molecular and immunological heterogeneity of eosinophilic esophagitis: Insights and subtyping
Source: PLoS One. 2026 Mar 12;21(3):e0342834. doi: 10.1371/journal.pone.0342834 (PMC12981482; doi:10.1371/journal.pone.0342834)

## Slide 1
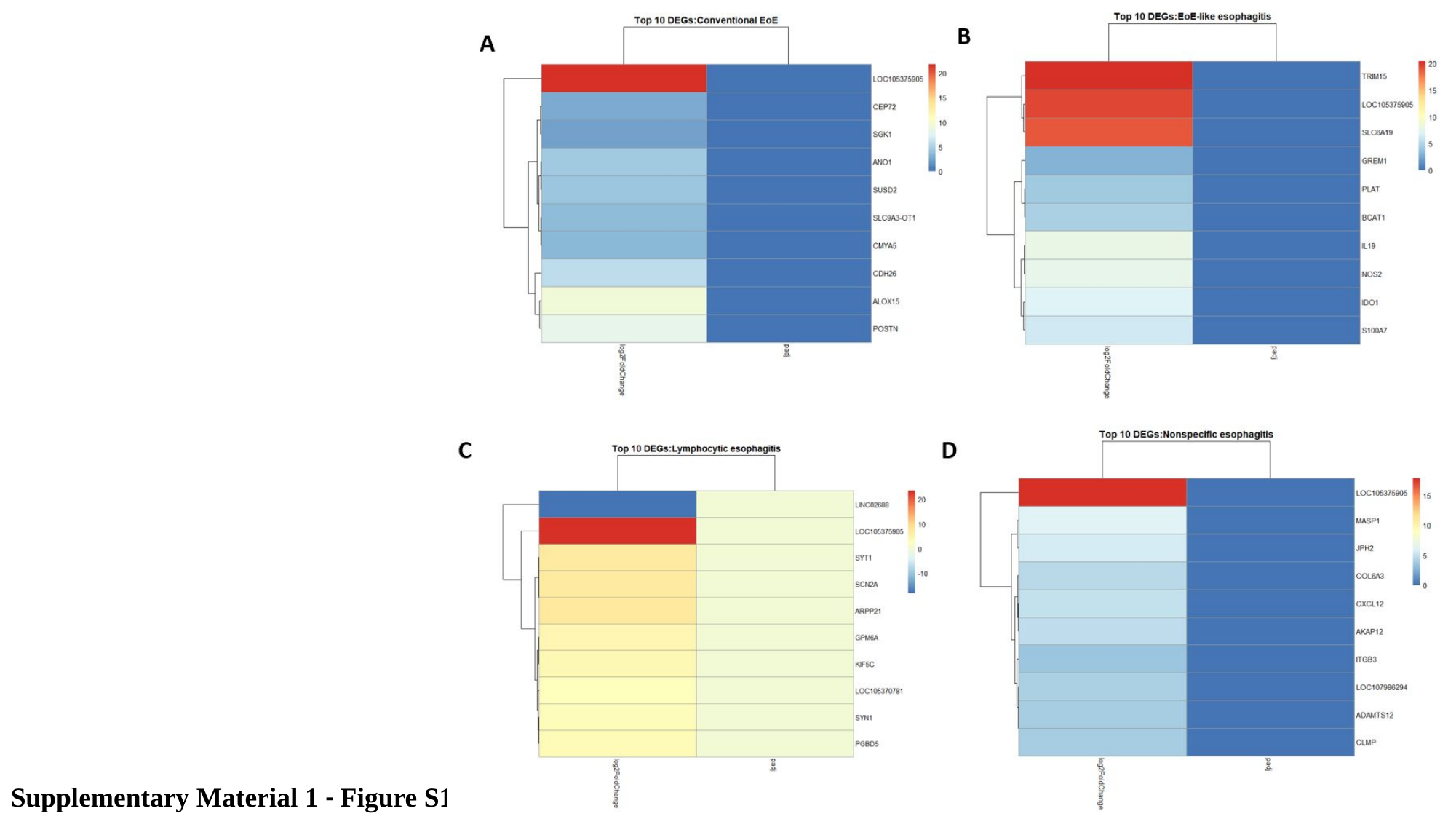

Supplementary Material 1 - Figure S1

Supplement: S1 Fig — The results highlight key genes POSTN1 as one of the key genes differentially upregulated in conventional EoE patients. (PPTX) [file pone.0342834.s001.pptx]

## Slide 1
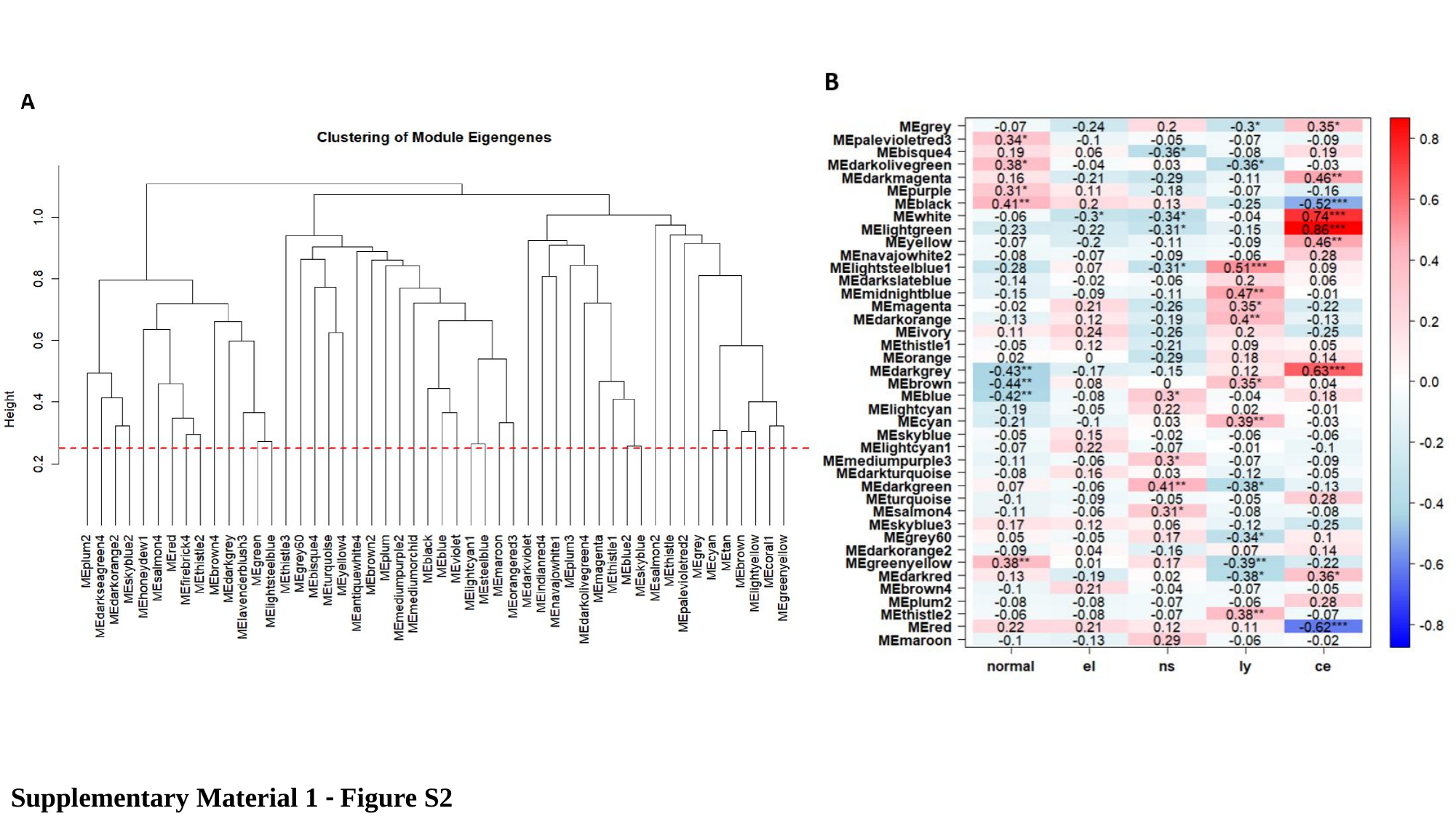

Supplementary Material 1 - Figure S2

## Slide 2
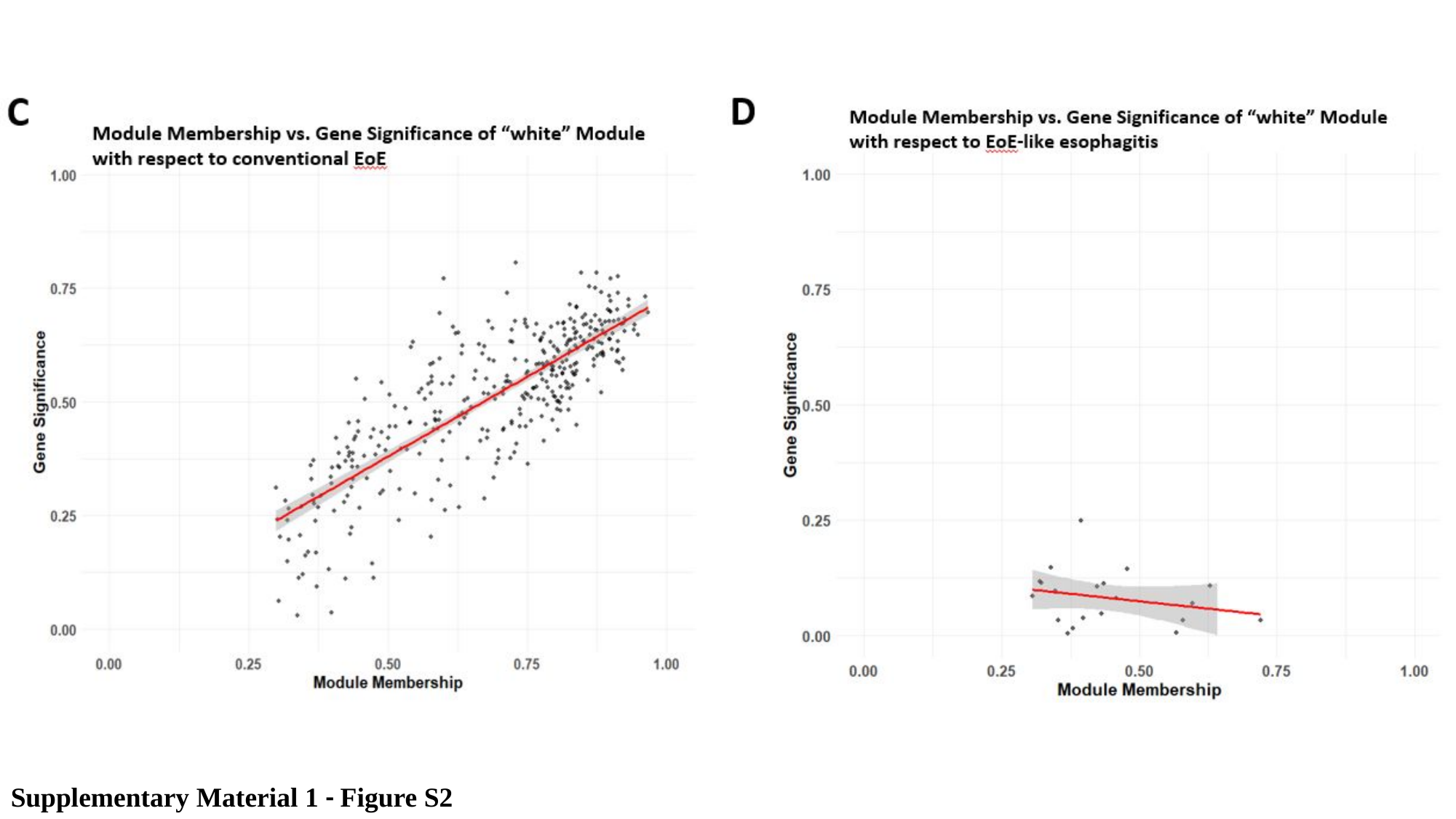

Supplementary Material 1 - Figure S2

Supplement: S2 Fig — (PPTX) [file pone.0342834.s002.pptx]

## Slide 1
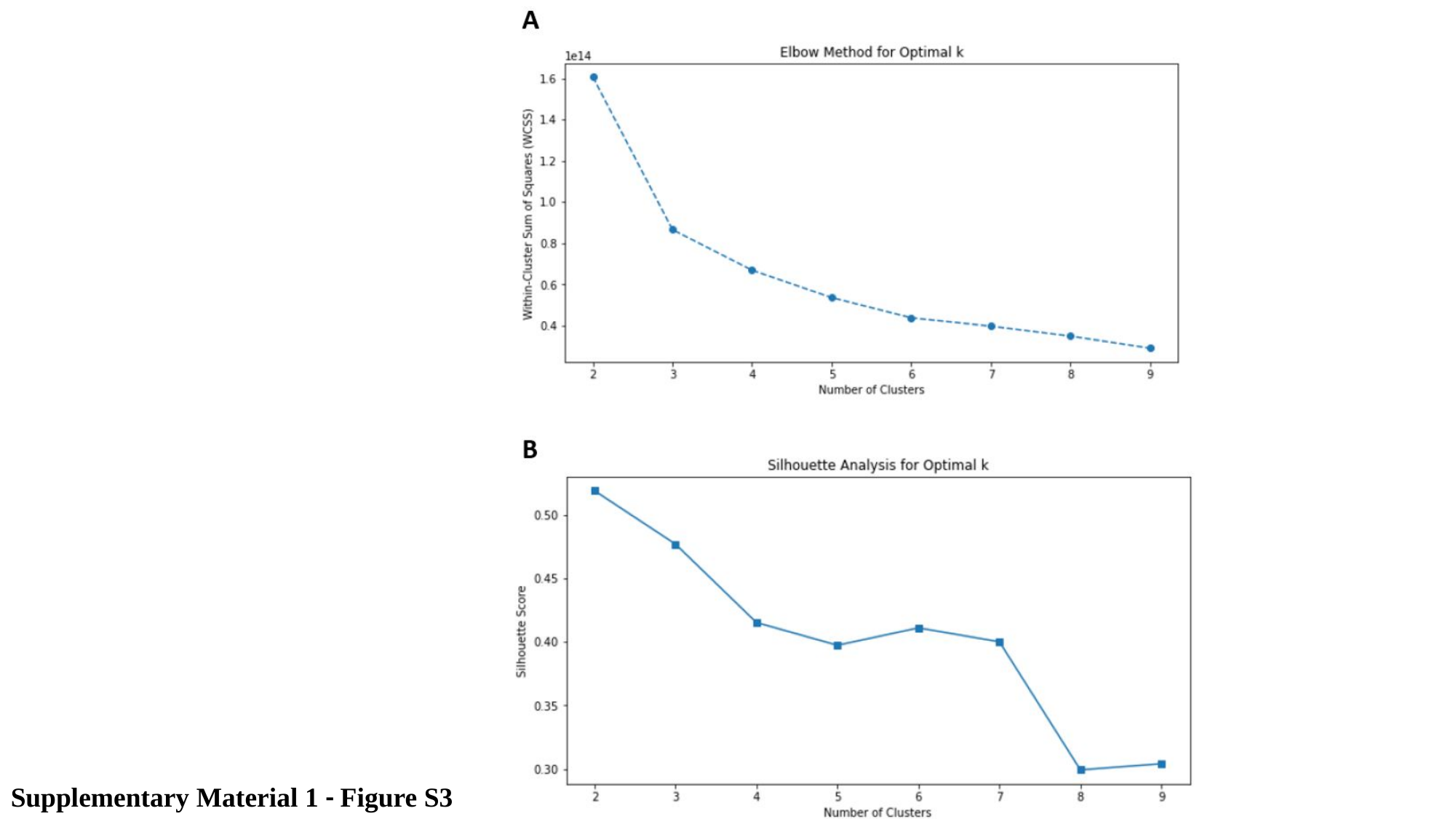

Supplementary Material 1 - Figure S3

Supplement: S3 Fig — The Elbow Method plots the within-cluster sum of squares (WCSS) against the number of clusters, with the “elbow” point indicating the optimal k. The Silhouette Analysis evaluates cluster separation, with higher silhouette scores reflecting better-defined clusters. Both methods suggested that three clusters provide the best representation of the dataset. (PPTX) [file pone.0342834.s003.pptx]

## Slide 1
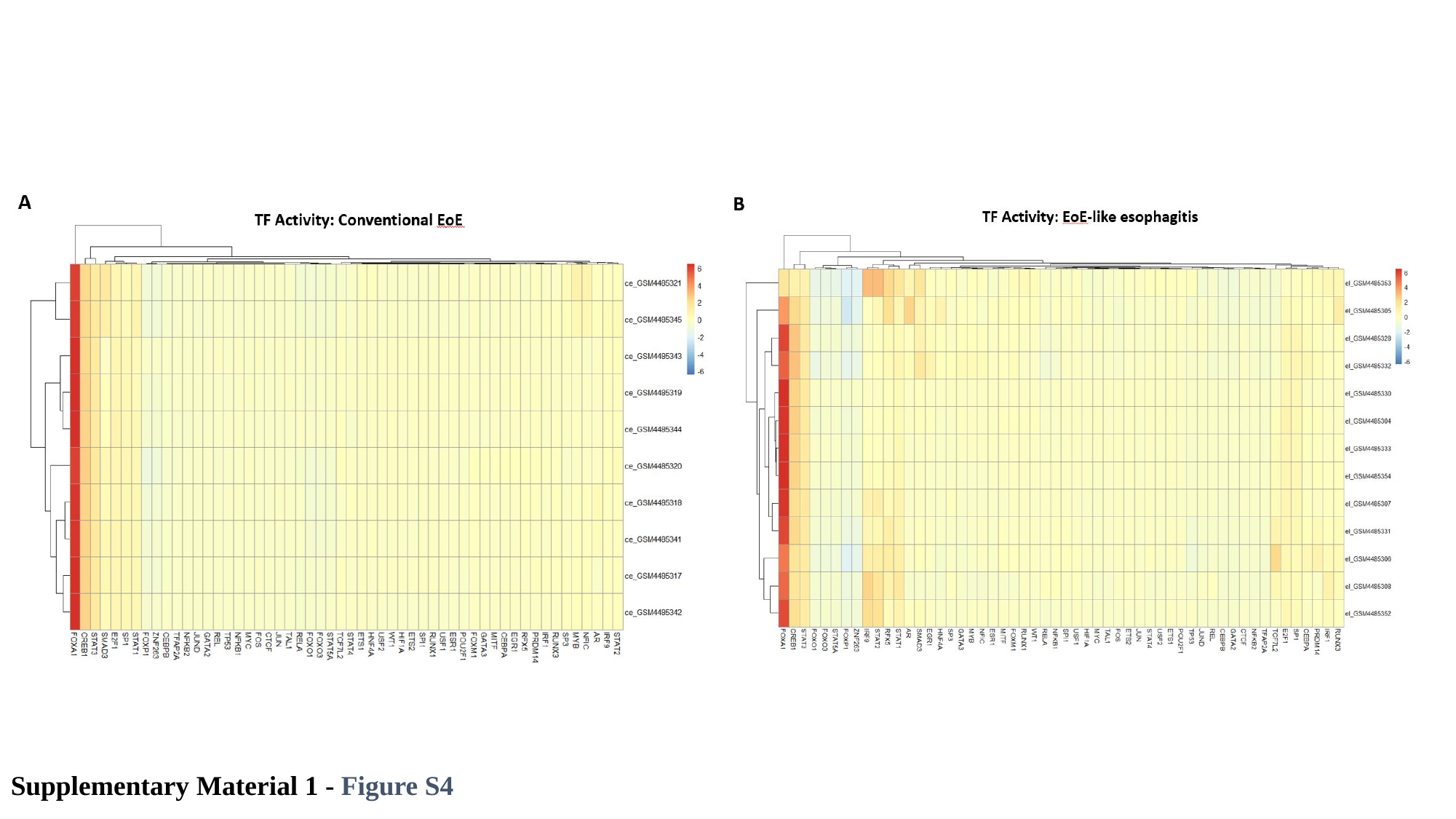

Supplementary Material 1 - Figure S4

## Slide 2
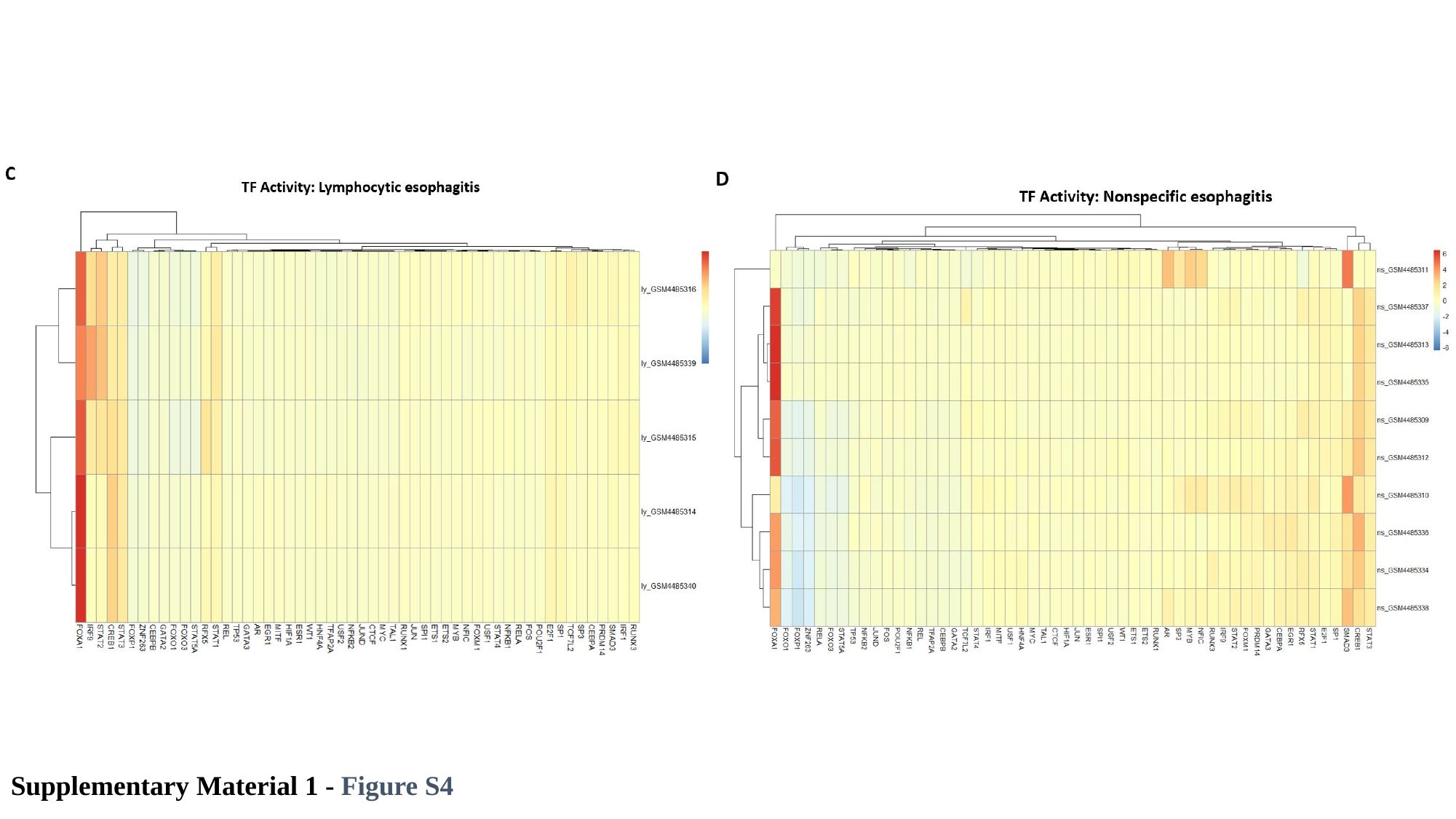

Supplementary Material 1 - Figure S4

Supplement: S4 Fig — The analysis identifies key transcription factors driving gene expression changes in each subtype, shedding light on potential regulatory networks contributing to disease pathology. (PPTX) [file pone.0342834.s004.pptx]

## Slide 1
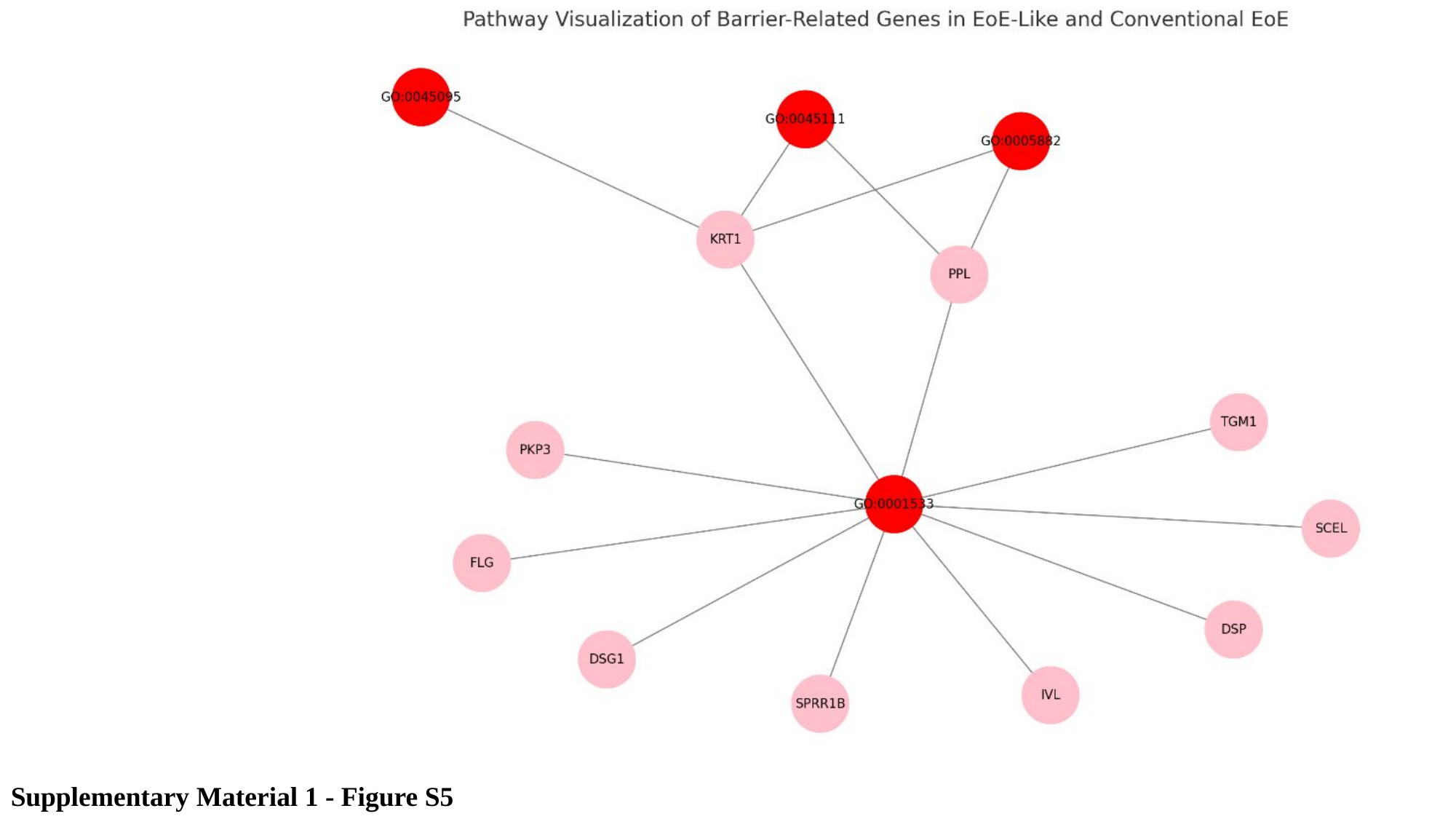

Supplementary Material 1 - Figure S5

Supplement: S5 Fig — Red nodes represent the GO terms (specific cellular components) enriched in Conventional EoE, while the pink nodes represent the associated genes linked to these GO terms. This is consistent with the findings that EoE-like esophagitis does not exhibit significant structural barrier dysfunction compared to Conventional EoE, which showed strong evidence of downregulation in components like the cornified envelope (GO:0001533) and associated genes (e.g., DSP, PKP3, TGM1, SCEL, SPRR1B, FLG). (PPTX) [file pone.0342834.s005.pptx]

## Slide 1
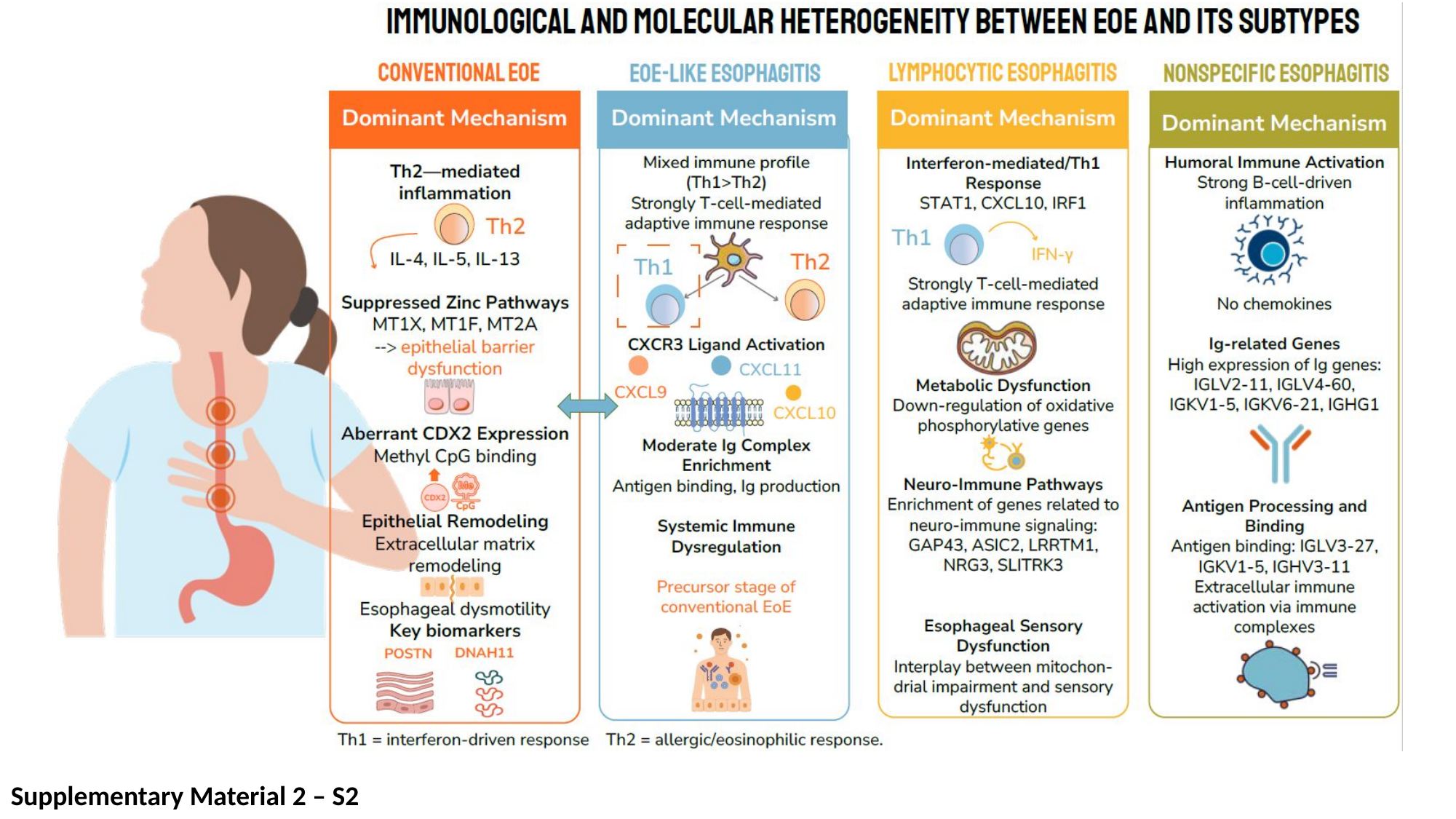

Supplementary Material 2 – S2

Supplement: S7 Fig — Simplified schematic summarizing the dominant immune pathways and molecular features identified for each subtype of esophagitis. Conventional EoE shows Th2-driven inflammation, zinc pathway suppression, and epithelial remodeling; EoE-like esophagitis exhibits a mixed Th1/Th2 immune profile and CXCR3-ligand signaling; lymphocytic esophagitis demonstrates interferon-mediated Th1 activation with mitochondrial and neuro-immune dysfunction; and nonspecific esophagitis is characterized by humoral immune activation with strong B-cell and immunoglobulin gene enrichment. (PPTX) [file pone.0342834.s007.pptx]
