# Supplementary material for: Molecular and immunological heterogeneity of eosinophilic esophagitis: Insights and subtyping
Source: PLoS One. 2026 Mar 12;21(3):e0342834. doi: 10.1371/journal.pone.0342834 (PMC12981482; doi:10.1371/journal.pone.0342834)
Supplement: S6 Fig — Tier 1 applies conventional histologic assessment, using the presence of ≥15 eosinophils per high-power field as the initial criterion for confirming conventional EoE. Tier 2 incorporates immune-axis profiling to identify Th2-dominant inflammation (↑IL4, IL5, IL13; ↓ MT1X, MT2A), supporting the diagnosis of conventional EoE even in borderline eosinophil counts. Tier 3 extends classification to include interferon-driven (STAT1, IRF1, CXCL10) or mixed Th1/Th2 (CXCR3 ligands) signatures, as well as humoral-immune enrichment (IGHV, IGKV) to distinguish lymphocytic, EoE-like, and nonspecific esophagitis subtypes. Candidate biomarkers such as POSTN, DNAH11, CXCL10, STAT1, and immunoglobulin-related transcripts (IGHV, IGKV) represent potential molecular markers for future clinical validation. This conceptual flowchart illustrates how the study’s subtype-specific molecular insights could be adapted into a diagnostic algorithm that bridges transcriptomic research with clinical decision-making. (PPTX) [file pone.0342834.s006.pptx]

## Slide 1
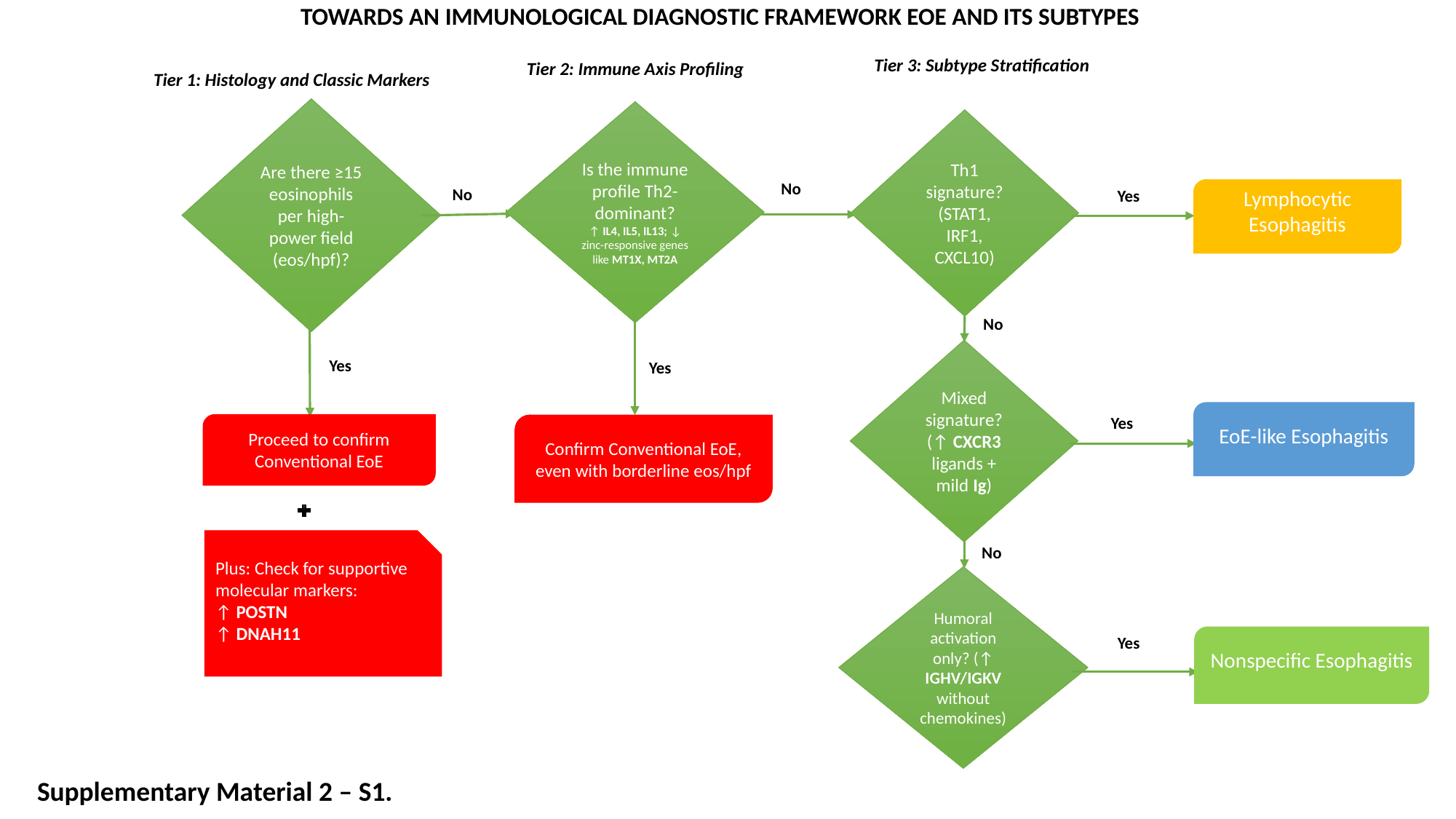

TOWARDS AN IMMUNOLOGICAL DIAGNOSTIC FRAMEWORK EOE AND ITS SUBTYPES
Tier 3: Subtype Stratification
Tier 2: Immune Axis Profiling
Tier 1: Histology and Classic Markers
Are there ≥15 eosinophils per high-power field (eos/hpf)?
Is the immune profile Th2-dominant?
↑ IL4, IL5, IL13; ↓ zinc-responsive genes like MT1X, MT2A
No
Yes
Yes
Proceed to confirm Conventional EoE
Confirm Conventional EoE, even with borderline eos/hpf
Plus: Check for supportive molecular markers:
↑ POSTN
↑ DNAH11
Th1 signature? (STAT1, IRF1, CXCL10)
No
Yes
Lymphocytic Esophagitis
No
Mixed signature? (↑ CXCR3 ligands + mild Ig)
EoE-like Esophagitis
Yes
No
Humoral activation only? (↑ IGHV/IGKV without chemokines)
Nonspecific Esophagitis
Yes
Supplementary Material 2 – S1.
