# Supplementary material for: Molecular and immunological heterogeneity of eosinophilic esophagitis: Insights and subtyping
Source: PLoS One. 2026 Mar 12;21(3):e0342834. doi: 10.1371/journal.pone.0342834 (PMC12981482; doi:10.1371/journal.pone.0342834)
Supplement: Attachment — Submitted filename: Comments from Reviewer3.docx [file pone.0342834.s009.docx]

Reviewer #3: Introduction:

**1. For conventional eosinophilic esophagitis (EoE) and its subtypes—EoE-like esophagitis, lymphocytic esophagitis, and nonspecific esophagitis, can authors demonstrate what are common features of immunology, genetics, and epigenetics?**

**Response:**

We thank the reviewer for this helpful suggestion. In response, we have expanded the Introduction to include a new paragraph summarizing the common immunological, genetic, and epigenetic features shared across EoE and its subtypes, with appropriate references added (see Introduction, paragraph 8). This addition highlights the unifying pathogenic framework across the disease spectrum.

**2. How do findings show molecular heterogeneity of esophageal inflammatory disorders? Can authors make more detailed descriptions?**

**Response:**

We thank you for this insightful suggestion. In the revised manuscript, we have expanded the Discussion (please see below) to include a new paragraph that points out how our findings demonstrate the molecular heterogeneity of esophageal inflammatory disorders. This section now describes the distinct transcriptional and regulatory features across conventional EoE, EoE-like, lymphocytic, and nonspecific esophagitis, complementing the shared immunological and barrier-related features already presented.

Overall, our findings also demonstrate the molecular heterogeneity of esophageal inflammatory disorders. Each subtype—conventional EoE, EoE-like, lymphocytic, and nonspecific esophagitis—exhibited distinct transcriptional and regulatory patterns, particularly in pathways governing immune activation, epithelial barrier integrity, and stress response. While conventional EoE was characterized by strong Th2 cytokine signatures, lymphocytic and nonspecific esophagitis displayed more variable immune networks, with enrichment of stress-related and metabolic pathways. EoE-like esophagitis further diverged by showing incomplete activation of canonical Th2-associated programs. These differences suggest that, although the disorders share overlapping immunopathogenic frameworks, they diverge at the molecular level in ways that likely influence clinical presentation and therapeutic response.

Methods:

**1. For Figure 1, do TF analysis, WGCNA, DE analysis and ML analysis share a parallel relationships? If not, can authors modify this figure?**

**Response:**

We thank the reviewer for this helpful comment. In our study design, TF activity inference, WGCNA, differential expression, and machine learning classification were all applied in parallel downstream of the Preprocessing and Quality Control step. Each represents an independent but complementary strategy to interrogate the same dataset. We have clarified this parallel relationship in the Methods (please language below) section to avoid any misinterpretation.

After preprocessing and quality control, four analyses—differential expression, weighted gene co-expression network analysis (WGCNA), transcription factor activity inference, and machine learning classification, were applied in parallel, each independently interrogating the same dataset.

**2. For Differential Gene Expression, why do authors select “Genes exhibiting |log2 fold change (FC)| > 1.5 and a p-adjusted value of p < 0.05 were considered statistically significant”?**

**Response:**

We thank the reviewer for this valuable comment. We selected |log2 fold change| > 1.5 and adjusted p-value < 0.05 as thresholds to ensure both statistical rigor and biological relevance. While other cutoffs (e.g., |log2FC| > 1.0 or > 2.0) could reasonably be applied, our choice represents a pragmatic balance, and helps capture a sufficient number of differentially expressed genes for downstream pathway and network analyses, while reducing the likelihood of including spurious changes. We have clarified this rationale in the Methods section and acknowledge that alternative thresholds may yield slightly different gene lists, though the overall biological interpretations are expected to remain consistent.

This cutoff was chosen to balance statistical confidence with biological relevance, ensuring robust gene-level changes for downstream analyses. While alternative thresholds could be applied, this choice provided a stable and interpretable gene set, consistent with prior transcriptomic studies (Mittal et al., 2025; Petrenko et al., 2024).

**3. For Gene Ontology and Functional Analysis, if it is a kind of ORA analysis, authors should indicate this point?**

**Response:**

We thank the reviewer for this suggestion. While we initially considered including an over-representation analysis (ORA) framework, our enrichment analyses were performed exclusively using Gene Set Enrichment Analysis (GSEA). We have revised the Methods to clarify this point. Please see language below.

To investigate the biological significance of key genes, we performed Gene Ontology (GO) and functional enrichment analyses using Gene Set Enrichment Analysis (GSEA), implemented via the R packages DOSE, org.Hs.eg.db, and enrichplot (Carlson, 2019; Yu, 2023; Yu et al., 2015).

**4. Do Dorothea and WGCNA share similar application conditions?**

**Response:**

Thank you for this question. The applications of DoRothEA and WGCNA differ in both scope and methodology. WGCNA is a network-based clustering framework used to identify modules of co-expressed genes, while DoRothEA is a knowledge-driven resource that, when combined with decoupleR, is used to infer transcription factor (TF) activity from expression data. Thus, while both approaches operate on the same transcriptomic dataset, they address different biological questions and do not share similar application conditions.

**5. “gene lists from the modules of interest into the STRING”, do they come from WGCNA analysis?**

**Response:**

We thank the reviewer for this helpful clarification. Yes, the gene lists uploaded to STRING were derived from modules of interest identified through WGCNA. We have revised the Methods section to specify this point explicitly. Please see below.

To construct the PPI of the modules of interest, we first inputted the gene lists from the WGCNA modules of interest into the STRING (https://string-db.org/) plugin in CytoScape to construct as protein-protein interaction (PPI) network.

**RESULTS:**

**1. Explain why are belt shape for A and C are different from B and D in Figure 2?**

**Response:**

We appreciate this observation. All volcano plots were generated using the same thresholds (|log2FC| > 1.5; padj < 0.05) and identical plotting parameters. The apparent differences in belt shape simply reflect the distribution of expression changes in each patient group relative to controls, rather than a methodological factor. Since the aim of the volcano plots is to highlight significantly up- and down-regulated genes, we did not focus on the overall contour of the plots in our interpretation. To clarify this, we have inserted the language below into the caption for Figure 2.

Note: Apparent differences in the contour (“belt shape”) of the plots reflect the inherent distribution of differential expression within each patient group, rather than methodological variation.

**2. “GO-BP revealed distinct clustering patterns and varying correlation levels”, can authors explain by disease mechanisms research progress?**

**Response:**

We thank the reviewer for this comment. We have clarified in the Results that the distinct GO-BP clustering patterns likely reflect differences in immune activation, epithelial barrier involvement, and tissue remodeling pathways across the esophagitis variants. Please see language below:

These clustering differences likely mirror distinct disease mechanisms, with variation in immune activation, epithelial barrier involvement, and tissue remodeling pathways shaping the molecular signatures of each subtype.

**3. What are common/different findings between DE-GO analysis and WGCNA-GO analysis?**

**Response:**

We thank the reviewer for this insightful comment. We have now added a comparison between DEG-based GO enrichment and WGCNA-derived GO analysis in the Results section. As described, DEG-GO highlights disease-specific effector processes, while WGCNA-GO emphasizes broader co-regulated immune and signaling hubs, with partial convergence at the gene level across subtypes. Please see language below:

To further integrate our DEG-GO gene enrichment with WGCNA-derived gene module lists, we compared results at the gene level. We observed partial convergence. In conventional EoE, inflammatory signaling genes such as FOS, JUN, DUSP1, and CXCL8 were identified by both DEG-GO and the lightgreen WGCNA module, whereas barrier integrity genes (IVL, LOR, SPRR1B, FLG) were specific to DEG analysis. In EoE-like esophagitis, antimicrobial defense genes (DEFB1, S100A8, S100A9, LCN2) overlapped between DEG-GO and the lightsteelblue1 module, while RNA catabolism genes (EXOSC3, DIS3, XRN1) were unique to DEG-GO. In lymphocytic esophagitis, chemokine and interferon-related genes (CXCL9, CXCL10, CXCL11, CCL5, STAT1) were consistently detected by both methods, whereas mitochondrial and translation-related DEGs (NDUFB2, NDUFS3, ATP5F1B, MRPL12) were captured only by DEG-GO. In contrast, nonspecific esophagitis showed limited overlap: immunoglobulin genes (IGHG1, IGKC, IGLC1, IGLV3-27, IGKV1-5) were strongly enriched in DEG-GO but were dispersed across modules and did not form co-expression clusters in WGCNA.

Taken together, DEG-GO analysis emphasizes disease-specific effector processes (such as epithelial barrier disruption, oxidative phosphorylation), whereas WGCNA-GO highlights coordinated immune signaling hubs such as chemokines.

**4. How are hub genes identified in PPI network by WGCNA module related with findings from DE analysis?**

**Response:**

We thank the reviewer for this suggestion. We cross-referenced hub genes identified within WGCNA-derived PPI networks with DEGs. Our analysis revealed strong convergence in conventional EoE, EoE-like esophagitis, and lymphocytic esophagitis, where key hub genes (e.g., FOS, JUN, CXCL8, S100A8, S100A9, STAT1) were also robust DEGs. In contrast, nonspecific esophagitis hub genes showed limited overlap with DEGs, reflecting diffuse immunoglobulin activity rather than tightly co-regulated hubs. These findings are now described in the Results section as follows:

To assess the relationship between WGCNA-derived hub genes and differential expression, we cross-referenced the hub genes identified within the PPI networks of WGCNA modules against DE analysis. We found substantial convergence: in conventional EoE, hub genes from the lightgreen module (e.g., *FOS, JUN, CXCL8*) were also significantly upregulated DEGs, reinforcing their central role in inflammatory signaling. In EoE-like esophagitis, hub genes such as *S100A8, S100A9*, and *LCN2* overlapped with DEGs, highlighting shared antimicrobial defense processes. In lymphocytic esophagitis, key interferon-inducible hub genes (*CXCL9, CXCL10, STAT1)* were also robust DEGs, supporting their role as drivers of Th1-skewed immunity. By contrast, in nonspecific esophagitis, many immunoglobulin DEGs (e.g., *IGHG1, IGKC, IGLC1*) did not emerge as WGCNA hubs, suggesting that antibody gene overexpression represents diffuse activity rather than tightly co-regulated network hubs.

**5. Have authors try unsupervised learning?**

**Response:**

Thank you for this question. Yes, we employed multiple unsupervised approaches in the study. Specifically, WGCNA represents an unsupervised clustering framework, grouping genes into co-expression modules without prior labels. In addition, we applied k-means clustering in our ML methodology which subsequently informed our supervised classification framework. We plan to explore these approaches in future work to validate and refine the subtype-specific molecular signatures identified here.

**6. Can results of TF analysis explain the results in DE/WGCNA analysis?**

**Response:**

We thank the reviewer for this suggestion. We have expanded the transcription factor activity section to explicitly relate TF findings to our DE and WGCNA analyses. Specifically, we now note that STAT3 and CREB1 were also detected as differentially expressed genes, whereas FOXA1, SMAD3, and TFAP2C showed regulatory activity without differential expression, highlighting both convergence and complementarity between these approaches. Please below:

Some of these TFs also appeared as differentially expressed genes, including *STAT3* in EoE-like and lymphocytic esophagitis and *CREB1* in lymphocytic esophagitis. In contrast, *FOXA1, SMAD3,* and *TFAP2C* showed significant inferred activity without corresponding differential expression, suggesting their regulation may occur post-transcriptionally or through upstream signaling.

DISCUSSION

**1. How are the immune mechanisms in EoE related with ECM remodeling, and cell motility and organ morphogenesis, especially for Th2-mediated inflammation?**

**Response:**

We agree with the reviewer and have expanded the Discussion to highlight how Th2-mediated inflammation (IL-13, IL-5, SMAD3 signaling) links to ECM remodeling, cell motility, and morphogenesis, supported by periostin upregulation and enrichment of motility-related pathways. Please added text below.

Importantly, in conventional EoE, Th2 cytokines (notably IL-13 and IL-5, with downstream SMAD3 signaling) connect inflammation to tissue remodeling. These signals drive eosinophil recruitment, epithelial injury, and activation of profibrotic programs, exemplified by periostin (POSTN) upregulation and ECM deposition. Enrichment of motility- and morphogenesis-related genes such as DNAH11 further suggests that inflammatory cues modulate epithelial turnover and cytoskeletal dynamics, contributing to barrier dysfunction, fibrosis, and structural remodeling.

**2. How are Epigenetic modifications involved in EoE? Are they connected to immune?**

**Response:**

We thank the reviewer for raising this point. As discussed in the Discussion section, we addressed the role of epigenetic modifications in EoE, highlighting CDX2 expression in association with methyl-CpG binding proteins, links to Th2-mediated inflammation, and parallels with Barrett’s esophagus. We also note the potential of epigenetic dysregulation to sustain epithelial remodeling and treatment resistance.

**3. Why is EoE-like esophagitis are different from cEoE, by mechanisms? How are about Lymphocytic Esophagitis, Nonspecific Esophagitis? Can authors link them together using your findings?**

**Response:**

We thank the reviewer for this suggestion. We have added a concise comparative synthesis highlighting how conventional EoE, EoE-like, lymphocytic, and nonspecific esophagitis differ mechanistically while sharing a common immune–epithelial axis. Please see inserted text below:

*Comparative mechanistic synthesis across esophagitis subtypes*

Our integrative analyses indicate that across the subtypes, a shared immune–epithelial axis emerges, but with distinct emphases. Conventional EoE is Th2-driven with barrier disruption and ECM remodeling, while EoE-like shows stronger innate activity and milder barrier changes. Lymphocytic esophagitis is marked by interferon-chemokine programs and metabolic stress, and nonspecific esophagitis by humoral signatures, supporting a spectrum model shaped by cytokine polarity, epithelial integrity, and metabolic state.

**4. For TF results, again, can authors link them together using your findings?**

**Response:**

We thank the reviewer for this suggestion. In the revised discussion, we now explicitly integrate transcription factor findings with our DEG and WGCNA results. Notably, TF activity (e.g., FOXA1, STAT3, SMAD3, STAT1/IRF1) reinforces the subtype-specific molecular programs, supporting a unifying regulatory axis that connects immune polarization, epithelial remodeling, and metabolic stress across esophagitis subtypes. Please see inserted text below:

Notably, transcription factor activity (e.g., FOXA1, STAT3, SMAD3, STAT1/IRF1) reinforced the subtype-specific patterns observed in DEG and WGCNA analyses, supporting a unifying regulatory axis that connects immune polarization, epithelial remodeling, and metabolic stress across esophagitis subtypes.
