# Supplementary material for: Molecular and immunological heterogeneity of eosinophilic esophagitis: Insights and subtyping
Source: PLoS One. 2026 Mar 12;21(3):e0342834. doi: 10.1371/journal.pone.0342834 (PMC12981482; doi:10.1371/journal.pone.0342834)
Supplement: Attachment — Submitted filename: Additional Comments from Reviewer4_Sep2025.docx [file pone.0342834.s010.docx]

**Review Comments to the Author**

Reviewer #4: The manuscript “Molecular and Immunological Heterogeneity of Eosinophilic Esophagitis: Insights and Subtyping” provides a comprehensive integrative multi-omics and bioinformatics approach to disentangling the heterogeneity of EoE and its related subtypes. The study is timely and addresses an important unmet need in gastroenterology and immunology, namely the lack of clear molecular criteria to differentiate overlapping esophageal inflammatory disorders.

Strengths:

- The integrative approach using gene expression profiling, WGCNA, enrichment studies, and machine learning provides robust cross-validation of findings.

- Identification of novel players such as DNAH11 and zinc pathway alterations adds originality and translational relevance.

- The clear delineation of subtype-specific pathways (e.g., immune dysregulation in EoE-like, mitochondrial impairment in lymphocytic esophagitis) is of high significance for potential precision medicine.

- The manuscript is generally well-written and logically structured.

**Author Response:**

We sincerely thank the reviewer for these generous and encouraging comments. We are pleased that the integrative multi-omics framework, cross-validation strategy, and subtype-specific analyses were recognized as key strengths of the study. We are grateful for the reviewer’s positive evaluation, which affirms the originality and translational potential of our work.

**Points for Improvement #1:**

- While analyses appear rigorous, the manuscript would benefit from clearer reporting of sample sizes, adjusted p-values, and validation across independent datasets where possible. This will strengthen confidence in subtype-specific conclusions.

**Author Response:**

We appreciate the reviewer’s thoughtful feedback and agree that clear reporting and validation are essential for transparency and reproducibility. The manuscript already provides detailed information on both sample sizes and adjusted p-values. Specifically, in the Data Collection section, we indicate that our analyses included 10 conventional EoE, 13 EoE-like esophagitis, 5 lymphocytic esophagitis, 10 nonspecific esophagitis, and 7 healthy control samples derived from the GSE148381 dataset. In the Methods (Differential Gene Expression) section, we specify that significance thresholds were based on Benjamini–Hochberg adjusted p-values (padj < 0.05) and |log₂ fold change| > 1.5. Based on your suggestion, we have added the following sentence to clarify the use of p-adjusted values:

All statistical significance values represent Benjamini–Hochberg–adjusted p-values (padj) to control for false-discovery rate across multiple comparisons.

Also, to improve visibility of this information, we have now restated the sample sizes in the Results section as follows:

DEG analysis was performed on a total of 45 esophageal biopsy samples, comprising 10 conventional EoE, 13 EoE-like esophagitis, 5 lymphocytic esophagitis, 10 nonspecific esophagitis, and 7 healthy control samples derived from the GSE148381 dataset.

Regarding validation, we agree that additional replication across independent datasets would further strengthen the study. However, GSE148381 is currently the only publicly available transcriptomic dataset that includes all four esophagitis subtypes— conventional, EoE-like, lymphocytic, and nonspecific— which limits opportunities for external validation at this time. To help address this, we applied several internal validation approaches—including WGCNA module–trait correlations, transcription factor activity inference, and Random Forest classification—which consistently reproduced the subtype-specific molecular signatures reported in the manuscript.

We have also added the following sentence to the Study Limitations section to acknowledge this context explicitly as follows:

While independent validation datasets for all esophagitis subtypes are currently unavailable, internal cross-validation within GSE148381 (via WGCNA, TF inference, and Random Forest modeling) provided convergent evidence supporting the robustness of identified subtype-specific markers.

**Points for Improvement #2:**

**- Pathway maps or schematic diagrams summarizing the distinct molecular landscapes of each subtype would enhance clarity for readers less familiar with systems biology.**

**Author Response:**

We sincerely thank the reviewer for this insightful suggestion. We agree that a clear visual summary improves accessibility for readers without a systems biology background. The manuscript originally included a detailed mechanistic overview in Supplementary Material 2 (Figure 1), which depicted the dominant molecular pathways for each esophagitis subtype. To further enhance clarity and reader engagement, we have updated this figure into a simplified schematic that more clearly summarizes the key biological processes, immune axes, and representative biomarkers for each subtype in a visually intuitive, lay-friendly format.

**Points for Improvement #3:**

**The connection between altered zinc homeostasis and epithelial dysfunction is compelling but currently speculative. Additional evidence or literature support should be added to avoid overinterpretation.**

**Author Response:**

We thank the reviewer for this helpful observation and fully agree that the relationship between zinc homeostasis and epithelial barrier integrity should be presented with appropriate caution. The manuscript already discusses this connection with supporting literature, citing key studies that describe the roles of metallothioneins and zinc transporters in mucosal integrity, oxidative-stress regulation, and epithelial repair (Jeong & Eide 2013; Ruttkay-Nedecky et al. 2013; Eussen et al. 2024). These citations provide mechanistic context for the transcriptomic signatures we observed in conventional EoE.

To further strengthen the mechanistic rationale, we have incorporated additional evidence showing that zinc status influences T-helper cell polarization, where zinc deficiency preferentially impairs Th1 cytokine production and biases toward a Th2-dominant immune response—a hallmark of conventional EoE (King et al. 2022; Wessels et al. 2022). We also added recent studies demonstrating that Th2/eosinophilic inflammation directly disrupts epithelial integrity through reduced claudin-7, suppressed HIF-1α, and dysregulated CD73/adenosine signaling (Nguyen et al. 2018; Masterson et al. 2019; Sridhar et al. 2025).

The relevant paragraph in the Discussion now reads:

Emerging evidence indicates that zinc status modulates T-helper cell polarization, with zinc deficiency preferentially impairing Th1 cytokine production and biasing immune balance toward Th2 inflammation (King et al., 2022; Wessels et al., 2022). Mechanistic studies further demonstrate that Th2/eosinophilic inflammation disrupts epithelial integrity in EoE through reduced claudin-7, suppressed HIF-1α, and dysregulated CD73/adenosine signaling (Nguyen et al., 2018; Masterson et al., 2019; Sridhar et al., 2025). This relationship is particularly relevant given recent evidence that zinc supplementation may reduce EoE severity (Eussen et al., 2024), suggesting that modulation of zinc homeostasis could represent a potential therapeutic strategy. While our transcriptomic results support a possible mechanistic link between zinc dysregulation and epithelial barrier impairment, further experimental validation will be needed to establish causality or therapeutic relevance.

Instead of:

This finding is particularly significant in light of recent studies suggesting that zinc supplementation can ameliorate EoE severity (Eussen et al., 2024). Hence, targeting zinc homeostasis through dietary interventions or pharmacological modulation of zinc transporters could represent a promising therapeutic avenue for conventional EoE.

These revisions provide updated mechanistic and immunological context, reinforce translational relevance, and ensure the interpretation remains appropriately balanced and evidence-based to avoid over-interpretation.

**Points for Improvement #4:**

**- Minor grammatical edits are needed (e.g., in the Results section, a few sentences are overly long and could be broken down for clarity).**

**Author Response:**

**We thanks the reviewer for the suggestion, we have made the following changes to the results section and broken down the sentences for clarity.**

**Original:** Based on preset criteria of an adjusted p-value < 0.05, all the patient groups showed differential genes expression from healthy controls.
**Split:** Using an adjusted p-value < 0.05, all patient groups showed differential gene expression relative to healthy controls. This confirms that each group diverges from the control state.

**Original:** These broad transcriptional shifts confirm that each esophagitis subtype has a distinct molecular profile, with both shared and unique gene expression patterns that likely reflect different underlying pathogenic mechanisms.
**Split:** These broad transcriptional shifts confirm that each esophagitis subtype has a distinct molecular profile. The profiles include shared and unique patterns, likely reflecting different underlying mechanisms.

**Original:** The suppression of these pathways suggests mitochondrial dysfunction and impaired energy metabolism in lymphocytic esophagitis, potentially impacting esophageal sensory and contractile function.
**Split:** Suppression of these pathways suggests mitochondrial dysfunction and impaired energy metabolism in lymphocytic esophagitis. These deficits could affect esophageal sensory and contractile function.

Original: Following the identification of the “MElightsteelblue1” and “MEwhite” gene modules as modules of interest for lymphocytic esophagitis and EoE-like esophagitis, we performed a GO enrichment analysis to uncover functionally enriched pathways associated these modules.

**Split & fix:** After identifying ‘MElightsteelblue1’ and ‘MEwhite’ as modules of interest, we performed GO enrichment analysis. This uncovered functionally enriched pathways associated **with** these modules.

**Original:** Taken together, DEG-GO analysis emphasizes disease-specific effector processes (such as epithelial barrier disruption, oxidative phosphorylation), whereas WGCNA-GO highlights coordinated immune signaling hubs such as chemokines.
**Split:** Taken together, DEG-GO emphasizes disease-specific effector processes (e.g., epithelial barrier disruption, oxidative phosphorylation). By contrast, WGCNA-GO highlights coordinated immune signaling hubs such as chemokines.

**Original:** Though traditional analytical approaches have provided valuable insights EoE pathophysiology, these techniques often fall short in capturing the intricate patterns and interactions within high-dimensional biological data.
**Split & fix missing ‘into’:** Traditional analytical approaches have provided valuable insights **into** EoE pathophysiology. However, they often miss intricate patterns and interactions in high-dimensional data.

Original: Inferring transcription factor (TF) activity is a critical step in understanding the regulatory mechanisms driving gene expression in different conditions, particularly in diseases with complex pathophysiology such as conventional EoE and related subtypes. We aimed to identify key regulatory factors that modulate gene expression, unraveling insights into potential therapeutic targets and pathways involved in disease progression.

**Split:** “Inferring transcription factor (TF) activity is critical for understanding regulatory mechanisms. This approach can also reveal potential therapeutic targets and pathways in disease progression.”

**Original:** These findings do not only reinforce the role of immune signaling and esophageal tissue remodeling in conventional EoE, but provide novel insights on molecular distinction between this condition and the EoE-like subtype, based the upregulation or downregulation of cell proliferation.
**Split & clean:** These findings reinforce the role of immune signaling and tissue remodeling in conventional EoE. They also provide new insight into how conventional and EoE-like subtypes differ, particularly in cell-proliferation programs.

**Original:** In patients with conventional, FOXA1 exhibited the highest activity across all samples, followed by CREB1 and STAT3, with consistent clustering observed among the samples (Fig 7B).”
**Split & fix missing word:** “In conventional EoE, FOXA1 showed the highest activity across samples, followed by CREB1 and STAT3. Clustering was consistent across individuals (Fig. 7B).

**Original:** In contrast, FOXA1, SMAD3, and TFAP2C showed significant inferred activity without corresponding differential expression, suggesting their regulation may occur post-transcriptionally or through upstream signaling.
**Split:** In contrast, FOXA1, SMAD3, and TFAP2C showed high inferred activity without differential expression. This suggests regulation at post-transcriptional or upstream signaling levels.

**Points for Improvement #5:**

**- The conclusion should emphasize how these findings could realistically be applied in clinical diagnostics or therapeutic development. A short section outlining potential biomarkers suitable for clinical validation would be valuable.**

We thank the reviewer for this insightful suggestion. We agree that emphasizing the translational significance of our findings enhances the manuscript’s clarity and impact. To address this, we have revised the Discussion under the subsection “Implications for Disease Classification and Treatment” to explicitly outline how our findings could inform clinical diagnostics and therapeutic development.

This section now introduces a tiered diagnostic schematic (Supplementary Material 2 – Figure 2) that integrates histopathologic evaluation, immune-axis profiling, and molecular stratification to demonstrate how subtype-specific signatures could be applied in clinical contexts. We highlight candidate biomarkers—including POSTN, DNAH11, CXCL10, and STAT1—as potential targets for future diagnostic validation and precision-guided treatment strategies.

Importantly, we have clarified that this framework is hypothesis-generating and will require prospective clinical validation before diagnostic implementation. These revisions ensure that the manuscript clearly communicates the translational potential of our results while maintaining appropriate scientific caution. The newly incorporated section paragraph is as follows:

To illustrate how these findings could be translated into a practical diagnostic context, we propose a tiered diagnostic schematic that integrates histopathologic evaluation, immune-axis profiling, and subtype-specific molecular markers (Supplementary Material 2, Figure 2). This framework proposes the use of candidate biomarkers such as *POSTN, DNAH11*, *CXCL10*, and *STAT1* to complement current histologic criteria to improve subtype differentiation and diagnostic accuracy. While this schematic offers a promising conceptual model, it should be regarded as hypothesis-generating, requiring validation in prospective clinical studies before any diagnostic application.

The overall conclusion has also been modified to include the following highlight:

The molecular and immunological distinctions identified between conventional EoE and its subtypes—together with the proposed diagnostic framework (Supplementary Material 2, Figure 2)— provide a foundation for refining diagnostic criteria, redefining disease classification, and guiding the development of targeted therapeutic strategies, including biomarker-driven diagnostics, immunomodulatory interventions, and metabolic therapies.
